# The Impact of Conscientiousness on Participant Drop-Out: A Novel Method for Estimating Missingness

**DOI:** 10.1093/geroni/igaa057.1286

**Published:** 2020-12-16

**Authors:** Tomiko Yoneda, Nathan Lewis, Jonathan Rush, Andrea Piccinin, Bryan James, Scott Hofer, Graciela Muniz Terrera

**Affiliations:** 1 University of Victoria, Victoria, British Columbia, Canada; 2 Psychology, Victoria, British Columbia, Canada; 3 Rush Alzheimer’s Disease Center, Chicago, Illinois, United States; 4 University of Edinburgh, University of Edinburgh, Scotland, United Kingdom

## Abstract

Individuals low in conscientiousness are typically characterized by higher rates of dropout in longitudinal studies compared to individuals high in conscientiousness. Given that low conscientiousness is associated with increased risk of mortality and several adverse health behaviours and outcomes, attrition of individuals low in conscientiousness may result in systematic bias particularly relevant to developmental research focused on morbidity and mortality in older adulthood. Further, methods commonly used to estimate missing data require monotone coding patterns and untestable assumptions (e.g., MAR), and do not typically account for death as a competing risk factor. This project analyzed data drawn from the Memory and Aging Project (N=1156; Mage=79.2 years; 76.1% female) using multistate survival models to estimate the impact of conscientiousness on transitions between study wave participation over time (i.e., response, non-response), and death. With conscientiousness measured at baseline and death status determined by death records, complete state data are available for each study wave, unlike methods commonly used to model and estimate missingness. Adjusting for age, sex, and education, analyses revealed that higher levels of conscientiousness are associated with decreased likelihood of transitioning to non-response (HR= 0.97, CI’s 0.95, 0.99) and death (HR=0.96, CI’s 0.93, 0.99). These results suggest that over-sampling individuals low in conscientiousness during study recruitment may be important to better represent the general population, particularly when data are collected over several years or decades. Discussion will focus on how systematic bias introduced by higher response rates of individuals high in conscientiousness may impact health-related research based on longitudinal data.

